# Criterion and Concurrent Validity of the activPAL™ Professional Physical Activity Monitor in Adolescent Females

**DOI:** 10.1371/journal.pone.0047633

**Published:** 2012-10-19

**Authors:** Kieran P. Dowd, Deirdre M. Harrington, Alan E. Donnelly

**Affiliations:** 1 Department of Physical Education and Sport Sciences, University of Limerick, Limerick, Ireland; 2 Pennington Biomedical Research Center, Baton Rouge, Louisiana, United States of America; University of Granada, Spain

## Abstract

**Background:**

The activPAL has been identified as an accurate and reliable measure of sedentary behaviour. However, only limited information is available on the accuracy of the activPAL activity count function as a measure of physical activity, while no unit calibration of the activPAL has been completed to date. This study aimed to investigate the criterion validity of the activPAL, examine the concurrent validity of the activPAL, and perform and validate a value calibration of the activPAL in an adolescent female population. The performance of the activPAL in estimating posture was also compared with sedentary thresholds used with the ActiGraph accelerometer.

**Methodologies:**

Thirty adolescent females (15 developmental; 15 cross-validation) aged 15–18 years performed 5 activities while wearing the activPAL, ActiGraph GT3X, and the Cosmed K4B2. A random coefficient statistics model examined the relationship between metabolic equivalent (MET) values and activPAL counts. Receiver operating characteristic analysis was used to determine activity thresholds and for cross-validation. The random coefficient statistics model showed a concordance correlation coefficient of 0.93 (standard error of the estimate = 1.13). An optimal moderate threshold of 2997 was determined using mixed regression, while an optimal vigorous threshold of 8229 was determined using receiver operating statistics. The activPAL count function demonstrated very high concurrent validity (r = 0.96, p<0.01) with the ActiGraph count function. Levels of agreement for sitting, standing, and stepping between direct observation and the activPAL and ActiGraph were 100%, 98.1%, 99.2% and 100%, 0%, 100%, respectively.

**Conclusions:**

These findings suggest that the activPAL is a valid, objective measurement tool that can be used for both the measurement of physical activity and sedentary behaviours in an adolescent female population.

## Introduction

Increased levels of moderate to vigorous physical activity (PA) have the potential to improve cardio-metabolic risk factors, improve bone health, reduce the risk of depression and reduce the risk of becoming overweight/obese in childhood, adolescence and in adulthood [1], [2]. Despite the widespread publication of the benefits of PA, levels remain low in many countries [3], [4]. Furthermore, the most significant decrease in levels of PA occur in later adolescence, with greater decreases observed in females [3], [5]. This is critical, as the processes associated with long term risk of diseases, such as coronary heart disease, begin in childhood and adolescence [6].

Advancing the field of free-living activity measurement requires the development of methodologies that are practical in habitual settings. These methodologies are crucial when relating levels of PA to indices of health 7,8. Over the past two decades, accelerometry has become the preferred method of objectively examining PA in free-living populations [9]–[11]. This is primarily due to the rich information obtained from the devices [10], [12], coupled by relatively high levels of reliability and validity and the lowering costs of the monitoring devices themselves [9]. Typically, accelerometers record raw accelerations, and proprietary algorithms calculate arbitrary units known as accelerometer or activity counts over a specified time period or epoch (e.g. 15 seconds). The most frequently employed method of examining these activity counts has been to classify them into PA levels (light, moderate, vigorous) using predetermined thresholds [12]. Total minutes spent per day at each level and frequency, intensity and duration of PA can then be calculated [7], [12].

In recent years, the quantification of sedentary behaviour, as well as PA, has become extremely topical, as the deleterious effects of sedentariness have been emphasised [13]. Inactivity physiologists have highlighted the negative effect of sedentary behaviours on indices of health in rats, and have suggested the loss of contractile stimulation of large skeletal muscles as one the major physiological variables which regulates muscle enzyme lipoprotein lipase (LPL) [14]–[16]. The suggestion that isometric contraction of antigravity muscles produce electromyographic and skeletal muscle LPL change [15], [16] implies that activities such as standing, which would previously have been considered sedentary, should now be considered as distinct activity behaviours [13]. Consequently, sedentary behaviour is now characterized by energy expenditure below 1.5 metabolic equivalents (METs) while in a sitting or lying position during waking hours [17]. To date, epidemiological evidence has supported the physiological observations, highlighting the negative effect of sedentary patterns and behaviours in both adolescents [18] and adults [19]. Unfortunately, existing methods used to examine sedentariness have significant limitations. Surrogate measures of sedentary behaviour, such as self-reported TV viewing time, do not accurately quantify sedentariness, and only examine one aspect of sedentary behaviour [20]. Furthermore, the use of indirect measures of sedentariness, such as the use of sedentary thresholds from accelerometer counts (e.g. ≤100 counts min^−1^) rely on the lack of ambulation or movement rather than directly measuring body position [21].

Due to the increasing interest in sedentary behaviour and the obvious interest in examining levels of PA, a device that is both a valid and reliable measure of both domains would be extremely valuable. While the ActiGraph GT1M and GT3X (Manufacturing Technologies Inc. Health Systems, Shalimar, FL), for example, are valid measures of PA, their measurement of sedentary behaviours are dependent on thresholds. It has been suggested that the use of such thresholds to determine sedentary time could lead to errors, as this analysis may include other activities, such as standing [13], [22], [23]. Recent technological developments have provided researchers with the tools to directly examine sedentary behaviours without the use of thresholds. The use of inclinometer-based activity monitor, such as the activPAL Professional Physical Activity Monitor (PAL Technologies Ltd., Glasgow, UK), has enabled researchers to directly identify periods of sitting/lying, standing and stepping, and have been encouraged for studies examining sedentary behaviours in detail [13], [24]. The activPAL (AP) is worn on the midline of the anterior aspect of the thigh. Due to this unique positioning, the inbuilt inclinometer is able to distinguish between sitting/lying and standing, while the activity monitoring function of the device allows the examination of ambulation, as with other existing devices. The device has been validated for the measurement of static and dynamic activities in adults [25], posture during free-living activities [26], step and cadence output in females [27] and in the examination of sedentary time in children [28] and adults [22]. While the AP has been used as a measure of habitual locomotion using steps [29], the activity count function has not been investigated in the same way as with the ActiGraph (AG) accelerometer, and activity counts have not been utilized to examine free-living PA in any population. Furthermore, the sit/lie and stand function has not been validated in an adolescent population.

The primary aims of this paper were to investigate the validity of AP activity counts in estimating energy expenditure, to perform a value-calibration of the AP and to validate thresholds for defining moderate physical activity (MPA) and vigorous physical activity (VPA) for an adolescent female population. This paper also aims to examine the concurrent validity of the AP by comparing activity counts across different activities with the AG accelerometer. Finally, the paper aims to compare the performance of the AP with the AG for estimating time spent sitting, standing and stepping.

## Methods

### Participants

Participants for the study were recruited from a community youth group in the West of Ireland. To be considered for inclusion in this study, participants had to be female, aged between 15 and 18 years and have no injury or illness that limited their ability to be physically active. This population were selected for investigation as female adolescents have been highlighted as a particularly inactive population, and as a result are of great interest to PA and health practitioners [3], [5], [30]. A local community youth group were approached to participate in this study. All female members of this group were invited to an information evening, where the study protocols and objectives were clearly outlined. All participants were provided with parental and participant information sheets and consent forms, and invited to return the completed consent forms at their next youth group meeting. All participants that returned completed participant and parental written informed consent were selected for participation (n = 40). Three participants from the original sample of 40 withdrew from the study. From the remaining participants, five data sets were excluded from analysis due to a malfunction of indirect calorimetry measurement. A total of 32 valid sets of VO_2_ data were obtained from the participants for statistical analysis. Two sets of AP data were not included in the analysis due to equipment malfunction, resulting in 30 full sets of AP, AG and simultaneous VO_2_ data for the current analysis. Each participant was allocated a number, and a randomization table was used to assign each participant to either an equation development group or a cross-validation group. The study was approved by the Faculty of Education and Health Sciences Research Ethics Committee at the University of Limerick.

### Physical Activity Measurement Devices

Physical activity was recorded using the activPAL™ Professional PA Monitor (Firmware: v 0.9.9) which is a single unit uniaxial accelerometer (53 x 35 x 7 mm) that weighs approximately 20 grams. The AP responds to gravitational accelerations resulting from segmental movement [31], and data is recorded at 10 Hz for each 15 second time interval (epoch). Proprietary algorithms provide outputs including time spent sitting/lying, standing, stepping, step counts, cadence and activity counts. The AP communicates with a Windows (Microsoft Corporation, Microsoft Excel 2010, One Microsoft Way, Redmond, WA, USA) compatible PC via a USB interface. The ActiGraph GT3X (Firmware v 4.1.0) (Pensacola, FL 32502, USA) is a small triaxial accelerometer (38 mm × 37 mm × 18 mm) that weighs approximately 27 grams. The device assesses acceleration in three individual orthogonal planes using a vertical axis, horizontal axis and a perpendicular axis. The AG samples accelerations at a rate of 30 Hz and the data can be reprocessed into epochs ranging from 1 to 60 seconds. The AG also communicates with a Windows compatible PC via a USB interface. For the purpose of this study, the AG monitor was initialized to record vertical accelerations every 15 seconds.

### Metabolic Unit

Oxygen consumption was measured breath-by-breath using a portable metabolic unit (Cosmed K4B^2^, Rome, Italy). The K4B^2^ is a lightweight system with a heart rate receiver. The Cosmed K4B^2^ has been deemed an appropriate criterion measure for minute-by-minute energy expenditure [12]. Over the duration of the study, the K4B^2^ was calibrated following standard manufacturer procedures. Before each testing session, the device was calibrated using known gas concentrations, and environmental conditions were updated. The VO_2_ data was downloaded and stored on a PC after each testing session.

### Testing Protocol

Participants arrived to the testing facility having fasted for two hours, refrained from smoking and drinking caffeine for 2 hours, and having refrained from MPA and VPA for 12 hours. Participants wore light shorts, t-shirt or vest, socks and running shoes. Height was measured without shoes and socks to the nearest 0.25 cm using a portable stadiometer (Seca model 214, Seca Ltd, Birmingham, UK) and weight was measured without shoes and socks to the nearest 0.1 kg using portable electronic scales (Seca model 770, Seca Ltd, Birmingham, UK). Body mass index was then calculated from the height and weight measures (kg/m^2^). After participants arrived to the test facility and had their height and weight obtained, participants were fitted with the AP, the AG and the Cosmed K4B^2^ metabolic unit. Both accelerometers were initialized prior to participant’s arrival and their internal clocks were automatically synchronised with the main investigator computer. To conform to the sampling time used by the AP, a 15 s^−1^ epoch was used for the AG. The AP was attached directly on the skin of the midline of the anterior aspect of the right thigh using a PAL*stickie,* (double sided hydrogel adhesive pad) and tube bandages were used as extra security to keep the activity monitor in place. The AG was worn around the waist on an elasticated band over the right hip bone. The metabolic unit was placed over their shoulders and a mask fitted over the face.

Participants were then introduced to the protocol of activities. Activities were completed in ascending intensity throughout the testing period. Participants were instructed when to start each activity and when to stop each activity by a single observer. The activity category, the exact start time and the exact finish time of each activity were recorded by the observer. Resting VO_2_ was measured for 25 minutes, to ensure the participants were provided an adequate amount of time to return to a rested state. During this time period, participants lay in a reclined position on a physiotherapy plinth in a darkened, quiet room. For the sitting activity, participants sat looking straightforward, placed their feet flat on the floor, placed their hands on their knees and were asked not to speak or take part in any other activities. For the standing activity, participants once again looked straightforward with their feet shoulder width apart, had their hands held by their side and were asked not to take part in any other activity. Participants did not lean against or hold on to any support while completing the seated and standing activities.

Participants then completed the 3 locomotor activities. Participants were asked to complete each activity at a pace that was comfortable to them, but within each speed range: slow walking (2.5–4.5 km.h^−1^), brisk walking (4.5–6.5 km.h^−1^) and light jogging (6.5–8.5 km.h^−1^). Prior to the study beginning, the upper and lower time limits required to complete each section of the track during each speed category were calculated. The time taken to complete each section of the track was then used to estimate the speed of each participant. During the first minute of measurement, the time it took for each participant to complete each section of the track was recorded. If participants completed each section too slowly or too quickly, they were deemed to be travelling too slow or too fast, and they were asked to adjust their speed accordingly. Once participants were comfortable at travelling within the speed category, the time required to complete each section of the track was recorded, and participants were encouraged to maintain this speed throughout the remainder of the activity. Feedback was provided to each participant throughout the remainder of each speed category in an attempt to maintain a relatively constant speed. This approach was used to simulate real-life activity and to reduce the clustering effect that set speeds may have during statistical analysis. Individual rest periods between each movement activity were used to return participants heart rate below 100 beats min^−1^. Once the protocol was completed, the collected data was downloaded to the main investigators PC.

### Calibration Activities

The activities included in the protocol were 1) resting VO_2_, 2) sitting on a chair, 3) standing upright unaided, 4) slow walking (2.5–4.5 km.h^−1^), 5) brisk walking (4.5–6.5 km.h^−1^) and 6) a light jogging (6.5–8.5 km.h^−1^). The activities included in the protocol were informed by a number of past studies which recommended sedentary and locomotor activities [24], [32], [33]. Other core activities which have been recommended for inclusion in validation and calibration studies, such as car driving, bicycling and stair ascending/descending [24] have not been included in this protocol. Car driving was not included for insurance reasons, bicycling was omitted as extremely low levels of participation in cycling have been observed in this population [34] and stair ascending/descending was not included for practicality reasons.

For the sitting and standing activities, data was collected for 5 minutes, while 7 minutes of data was collected for ambulatory activities, with the mean value of the final two minutes of each activity used for data analysis. These durations were selected as VO_2_ remains stable (at a steady state) after 3 minutes for light activity and after 3–5 minutes for more intense activities [35]. The internal time clocks of the AG, AP and metabolic unit were synchronized. The mean value of the final two minutes of each activity (excluding resting VO_2_) was used for data analysis, as participants were deemed to be at steady state energy expenditure during this period [32].

### Data Processing

Once data from both the AP and AG were downloaded, files were processed using the AP (v 5.9.1.1) and AG (ActiLife v4.4.1) software. For the AG, only accelerations measured in the vertical plane were used for comparative analysis and the low frequency extension was not employed. This created time stamped 15 sec^−1^ epoch-by-epoch information. Using the K4B^2^ software, the VO_2_ information was averaged for every 15 sec^−1^ period, synchronizing the start time with the protocol start time. The breath-by-breath VO_2_ data from the K4B^2^ and the resulting epoch-by-epoch data from both the AP and the AG were collated, ensuring that the protocol start time for each individual was synchronized for all three devices. The information recorded by the single observer for the activity categories was synchronized with the VO_2_, AP and AG data. The final 2 minutes of each activity performed were selected for analysis, and all data exported to Predictive Analytic Software (PASW) version 18.0 for Windows (SPSS Inc., Chicago, IL, USA).

When preparing data for examining the levels of agreement between sitting/lying, standing and stepping, the inclinometer function of the AP was used to examine sitting/lying time and standing time, while the accelerometer function of the AP was used to examine stepping time. A sedentary threshold of <100 counts min^−1^ has been suggested for use with the AG in youths from studies which have examined levels of agreement between the AP and the AG [28]. For this reason, a sedentary threshold of <100 counts min^−1^ was used to quantify sedentary time using the AG only. This information was compared with the recorded activity categories from the single observer.

### Statistical Analysis

The use of conventional 1 MET values is discouraged for use in both children and adults [36]. To normalise energy cost between participants during different tasks, participant’s individual resting metabolic rate (RMR) were used to calculate their resting MET values, with energy cost during activity being expressed in calculated METs (MET score = Activity VO_2_ mL·kg^−1^ min^−1^/Resting VO_2_ mL kg^−1^ min^−1^). The accelerometer data was plotted to coincide with the steady state VO_2_ for each of the performed activities. Spearman rho correlation coefficients were calculated between accelerometer output from both the AP and the AG and VO_2_ with an r value of >0.7 considered highly correlated.

A random coefficients statistical model, which accounts for repeated measures taken from the same participants, was used to examine the relationship between MET values (dependant variable) and the AP counts using the equation development group only. The concordance correlation coefficient (CCC = the mixed model equivalent of R^2^ in linear regression) was used to assess the goodness of fit of the equation [37], and this was presented with the Standard Error Estimate (SEE). Age, height and weight were not found to contribute to the fit of the model. Receiver operating characteristic (ROC) curves and analysis were also used to calculate an area under the curve (AUC) and define a threshold which optimizes sensitivity (correctly identified points at or above the activity intensity threshold) and specificity (correctly excluded activities below the activity intensity thresholds) [38]. It has been suggested that the use of decision boundaries and ROC analysis provides a method of determining accelerometer intensity thresholds with less misclassification than the previously employed regression formulae [39]. The intensity thresholds were developed using both mixed regression (MR) and ROC analysis. Both sets of thresholds were cross-validated using ROC analysis on an independent group. Sensitivity, specificity and area under the curve were examined and interpreted [40], and the optimal value for MPA and VPA were identified. All analyses were undertaken using PASW statistics.

A non-parametric Spearman correlation was performed to examine the concurrent validity of the count·15 s^−1^ function of the AP with that of the AG. To examine the validity of the AP when classifying sitting, standing and stepping time, data from both the AP and the AG were compared with activity information recorded by the single observer. Agreement between both the AP and AG and direct observation was examined on a minute-by-minute basis. This entailed observing the amount of time each participant spent in each activity, and examining how well this time agreed with the observed activity category. Percentage Agreement, Sensitivity and Predictive Values were calculated [26]. Percentage agreement is defined as the agreement between all observed samples and activity monitoring samples ((number of observed samples which were correctly identified by AP or AG * 100)/total number of samples). Sensitivity was defined as the degree to which the activity monitors correctly detected the activity category ((number of observed samples which were correctly identified by AP or AG for each activity category * 100)/total number of samples for each activity category). Predictive Value was defined as the level to which each activity monitor determined category agreed with the observed activity category ((number of matching samples between observed values and AP or AG for AP or AG activity category * 100)/total number of samples for AP or AG activity category).

## Results

Participants mean age was 17.2 yrs. (±0.9), mean height was 1.7 m (±0.1), mean weight was 65.4 kg (±9.2) and the mean body mass index of the population was 23.2 kg·m^−2^ (±2.8). There were no significant differences observed for age, height, weight or BMI between the development group and the cross-validation group.

Table 1 describes the mean and SD of VO_2_, MET scores, speeds and both AP and AG activity counts expressed in count·15 s^−1^ from the development group. The chosen activities provided a wide range of accelerometer counts (AP Range: 0 to 14198 counts·15 sec^−1^; AG Range: 0 to 3378 counts·15 sec^−1^) and MET scores (Range: 0.74 to 14.95 METs).

**Table 1:** Mean and SDs of VO_2_ ml·kg^−1^ min^−1^, MET scores, speed and both activPAL and ActiGraph activity counts.

Activity	VO_2_(ml·kg^−1^ min^−1^)	METs	activPAL counts·15 s^−1^(n = 15)	ActiGraph counts·15 s^−1^(n = 15)	Speed(km/h)
**Sitting**	4.2 (1.2)	1.1 (0.2)	5 (8)	0 (1)	N/A
**Standing**	4.3 (1.2)	1.1 (0.2)	15 (22)	1 (2)	N/A
**Slow Walk**	11.0 (1.9)	3.0 (0.7)	3098 (858)	632 (174)	3.6 (0.4)
**Brisk Walk**	14.3 (2.1)	3.9 (0.8)	5011 (869)	940 (156)	4.9 (0.4)
**Light Jogging**	31.1 (4.5)	8.5 (1.9)	11086 (1624)	2368 (406)	7.3 (0.5)

The criterion validity of the AP was examined using VO_2_ as the criterion measure. The MR equation (N = 15) for the AP was developed for this population to correspond to activity categories (Moderate = 3–5.99 METs; Vigorous = 6 METs or greater) that have been recommended for examination in previously published literature. The developed equation is:
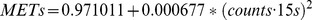



The concordance correlation coefficient (CCC) for counts·15 s^−1^ was identified as 0.93 (SEE = 1.20). When solving the equation, 2997 counts·15 s^−1^ was the value for a moderate threshold of 3 METs, while 7428 counts·15 s^−1^ was the value for a vigorous threshold of 6 METs.

### Mixed Regression Analysis

The ability of the MR to predict MET values from activity counts in an independent group was examined. The correlation values from this analysis are presented in Table 2. The mean absolute difference between actual and predicted MET values for non-locomotor activities, locomotor activities and all activities are also included in Table 2.

**Table 2:** Concordance correlation results comparing actual MET values with activPAL counts·15 s^−1^ based predicted MET values for all activities, non-locomotor activities (NLA) and locomotor activities (LA) in the cross-validation group (N = 15) (^*^p<0.01).

	ActualMean	PredictedMean	Mean Absolute Difference	SEE	r value
**All activities**	4.19 (3.16)	4.02 (3.04)	1.32	0.86	0.93^*^
**Non-locomotor Activities**	1.17±0.21	0.99±0.02	0.92	0.27	0.25
**Locomotor Activities**	5.92±2.74	5.75±2.5	1.55	1.06	0.87^*^

### ROC Analysis

The development group data was examined using ROC analysis, and revealed an AUC of 0.98 for MPA and 0.99 for VPA. For MPA, a threshold of 3329 counts·15 s^−1^ optimized sensitivity (0.93) and specificity (0.93), while a threshold of 8229 counts·15 s^−1^ optimized sensitivity (0.98) and specificity (0.97) for VPA. These values would be considered highly accurate [40].

### Cross-Validation of Developed Thresholds

The MPA and VPA thresholds from both MR analysis and ROC analysis were cross-validated, and the cross-validation results are presented in Table 3. Each threshold demonstrated high levels of sensitivity and specificity when cross-validated. As the AUC for both MPA thresholds and both VPA thresholds were the same, sensitivity and specificity were summed and the threshold with the highest value was selected as the optimal threshold. An optimal threshold of 2997 counts·15 s^−1^ was identified for MPA, while an optimal threshold of 8229 counts·15 s^−1^ was identified for VPA. The MPA threshold was developed using MR, and optimized sensitivity (95.7%) and specificity (94.5%). The VPA threshold was developed using ROC analysis, and optimized sensitivity (97.7%) and specificity (100%).

**Table 3:** Cross-validation results for the activPAL for sensitivity and specificity values for activity intensity thresholds developed using both mixed regression analysis and receiver operating characteristic (ROC) analysis.

	MPA		VPA	
	MixedRegression	ROC	MixedRegression	ROC
**Counts·15 s** ^−**1**^	2997	3329	7428	8229
**Sensitivity (%)**	95.7	91.3	97.7	97.7
**Specificity (%)**	94.5	95.9	99.2	100
**AUC**	0.99	0.99	1.0	1.0

### Concurrent Validity

**Figure 1:**
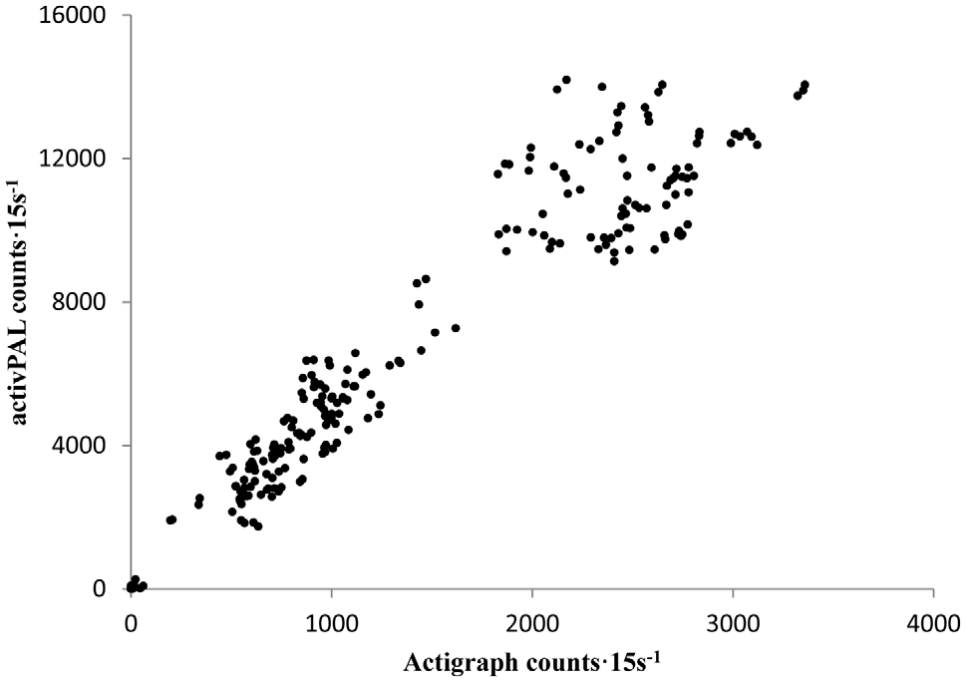
Relationship between the activPAL and ActiGraph GT3X count functions across all activities (N = 30).

There was a strong and positive relationship between the count function of the AP and that of the AG (r = 0.96, p<0.01) demonstrating very high concurrent validity between the two devices. Figure 1 presents the relationship between the count function of the AP and AG.

### activPAL and ActiGraph GT3X Sitting/Standing/Slow Walking vs. Direct Observation

**Figure 2:**
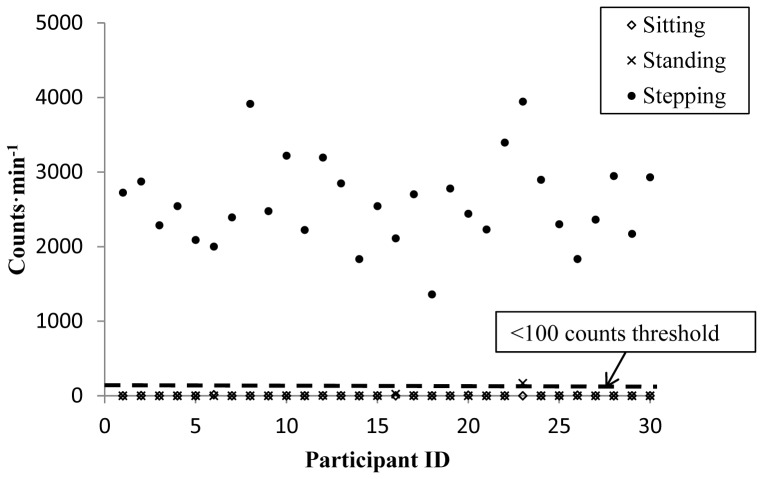
Sitting, standing and slow walking from direct observation compared with the <100 counts•min^−1^ ActiGraph sedentary threshold.

The results of the minute-by-minute analysis for levels of agreement, sensitivity and predictive values of the AP and the AG with direct observation for sitting, standing upright and slow walking are presented in Table 4. The overall levels of agreement between direct observation and AP for correct classification of sitting, standing and slow walking was 99.1%, while the overall agreement between direct observation and the AG was 66.7%. Additionally, a graphical representation of the activity counts min^−1^ for AG recorded sitting, standing and light stepping activities are presented and compared with the <100 counts min^−1^ sedentary threshold in Figure 2.

**Table 4:** Comparison of activPAL and ActiGraph determined sitting, standing and stepping with observed activity category.

	All activities	Sitting		Standing		Slow Walking	
	Agreement	S %	PV %	S %	PV %	S %	PV %
**activPAL**	99.1%	100%	100%	98.1%	100%	99.2%	100%
**ActiGraph**	66.7%	100%	100%	0%	0%	100%	100%

S = Sensitivity; PV = Predictive Value.

## Discussion

The AP demonstrated high levels of criterion validity, identifying a mixed model equivalent to an R^2^ of 0.93 (SEE = 1.20) when compared with METs across 7 different activity intensities. Threshold of 2997 and 8229 counts·15 s^−1^ for MPA and VPA identified optimum levels of sensitivity and specificity after cross validation in an independent sample. The activity count function of the AP demonstrated high levels of concurrent validity with the AG accelerometer count function (r = 0.96, p<0.01), while the AP was more accurate at distinguishing between sitting, standing and stepping than the sedentary thresholds employed when using the AG.

This study is the first study to develop count values that correspond to different activity intensities for the AP, which are typically used when examining PA in both observation and intervention research. Thus far, the literature on the use of the AP to examine PA is limited to sedentary and step-based measurements in laboratory [26], [31] and habitual settings [29]. While the existing AP software provides an easy to understand output of steps and cadence, which have been compared to MET estimates, it has been suggested that the use of activity counts in the examination of free living PA may be more applicable than the use of steps [7], [27]. To date, only one study has attempted to validate the AP with a criterion measure [27]. In this paper, Harrington et al. highlighted that the relationship between counts and measured METs was stronger than the relationship between steps and measured METs [27]. Similarly, the results presented in the present paper indicate that AP counts are highly associated with MET scores in an adolescent female population while performing a range of everyday activities (CCC = 0.93; SEE = 1.20). Weak correlations were observed when the MR predicted MET values from activity counts for non-locomotor activities (r = 0.25). However, the AP separately and accurately distinguishes between sitting and standing using an inbuilt inclinometer, which is more likely to be employed when examining non-locomotor activities in free living investigations. Although the AP employs a separate mechanism to determine sitting/lying and standing activities, non-locomotor activities are included to comply with recommendations for calibration and validation [12], [33]. The MR predicted MET values from activity counts within 1.32 METs, while a strong and significant correlation existed between actual and predicted MET values across all activities (r = 0.93; p<0.01). Similar to findings from other validation studies [7], [11], [32], these results suggest that the MR appears to be comparable when examining PA in this specific population.

Methodological differences in estimating intensity thresholds may have a substantial effect on resulting values [41]. It has been suggested that the use of linear and non-linear regression equations when developing activity intensity thresholds have significant limitations [39]. An alternate method for the development of activity intensity thresholds for use in accelerometer-based research has been developed [39] and implemented in studies with children and adolescents across a range of accelerometers [8], [11]. Through attempting to maximise the AUC, the ROC method of determining intensity thresholds places an equal emphasis on the importance of both sensitivity and specificity [11]. The use of ROC analysis and AUC in the development of intensity thresholds maximizes the sensitivity and specificity in classifying MET values correctly and reduces the error of estimating the true intensity [39]. In this paper, intensity thresholds were developed using both MR and ROC analysis, and a decision on which threshold was recommended for use was based on the sensitivity, specificity and area under the curve after cross-validation. When cross-validated with an independent sample, a MR determined threshold of 2997 counts·15 s^−1^ was identified for MPA (Sens = 95.7; Spec = 94.5; AUC 0.99), while an ROC determined threshold of 8229 counts·15 s^−1^ was identified for VPA (Sens = 97.7; Spec = 100; AUC 1.0). The observed high levels of sensitivity, specificity and AUC support the use of the developed threshold values within this specific population.

To date, only one study has compared the concurrent validity of the step count function of the AP and the step count function of the AG GT1M to video recorded steps [27], while no study has previously examined the concurrent validity of the activity count function of the AP with that of the AG. The findings of Harrington et al. (2011) identified that the AP step function was reasonably accurate at measuring moderate walking speeds, and was more accurate at measuring slow walking speeds when compared to the AG. The results presented in this paper have identified that the activity counts·15 s^−1^ function of the AP demonstrated very high concurrent validity when compared across all activities with the AG (r = 0.96; p<0.01). This would suggest that the AP is at least as effective in measuring locomotor activities as the uniaxial function of the AG.

Until now, large scale studies have utilized a sedentary count threshold (e.g. <100 counts min^−1^) when using the AG to define sedentary or sitting time [19], [30]. Researchers have examined the effectiveness of using a range of count thresholds for the AG compared with the AP (criterion measure) for examining sedentary time in youth. A sedentary threshold of <100 counts min^−1^ has now been suggested in this population [28]. Although the use of such sedentary thresholds may be appropriate for specific sedentary variables or research questions, whereby standing is considered a sedentary activity, the ability of such devices to examine specific sedentary patterns and behaviours is limited. It has been highlighted that standing is a distinct behaviour from sitting and can confer its own physiological benefits [13], [15], [16]. This paper has highlighted the accuracy of the <100 counts min^−1^ in estimating sitting/lying time (100%) and stepping time (100%). However, the inability of the AG to determine standing time has also been demonstrated (accuracy of 0%). These findings suggest that the use of a <100 AG counts min^−1^ in an adolescent female population will include time spent standing still, and highlights a limitation to the use of the <100 AG counts min^−1^ to determine sitting/lying patterns. This <100 counts min^−1^ threshold was applied to the AG vertical axis only as this threshold was developed using the vertical plane only from an older AG model. Findings of this study have also identified high levels of agreement between direct observation and the AP for sitting/lying (100%), standing (98.1%) and stepping (99.2%). Although the AP also records counts that can be examined using sedentary thresholds (similar to the AG), the AP employs an inbuilt inclinometer, which allows inclination of the thigh to be classified into sitting/lying or standing without the user resorting to using thresholds. The inclinometer function of the AP has previously been identified as both a valid and reliable measure of posture [26]. Devices, such as the AP, have provided researchers with the capability of directly examining sitting/lying behaviours and standing behaviour, while also examining levels of PA. The use of a single activity monitor that can distinguish between sitting/lying and standing time while also examining PA behaviours in a habitual setting has substantial potential in population-based research. The use of inclinometer-based activity monitors has substantial potential in the area of PA and health related research, as results from these monitors enable researchers to make stronger and more accurate associations between activity variables (including sitting/lying, standing and PA behaviours) and health variables [13], [24].

This paper has aimed to explore the effectiveness of the commonly employed AG (used in uniaxial mode) and the newer AP in measurement of PA and sedentary behaviour. The results have provided evidence that the AP can be used to examine standing, MPA and VPA as well as a measure of sedentary behaviour (for which it was originally developed). However, to recommend the AP for PA measurement alone would be premature, as the present study is only the first to validate the AP count function and to create threshold for MPA and VPA, compared with over 2 decades of validation and calibration work on the AG. However, the limitations of the AG for measuring sedentary behaviour cannot be overlooked. Our findings, particularly data presented in Figure 2, highlight the inability of the <100 counts min^−1^ sedentary threshold of the AG to differentiate between sitting and standing. The obvious approach would be to use both AG for measurement of PA and AP for the examination of sedentary behaviour. However, the potential cost and burden of wearing two devices on participants, especially children, cannot be overlooked as it may affect compliance. To our knowledge, only one study has examined levels of PA and sedentary behaviour using both the AP and AG devices [42]. Three days of valid measurement, which is below the recommended measurement period [9], were required from each participant, and insufficient data was provided by 28% of participants [42]. Published findings on the measurement of free living PA in adolescents using the AP only have identified high levels of compliance (8% of participants providing less than 4 valid days of measurement) [43]. Newer devices, such as the AG GT3X+, have incorporated inclinometer functions into existing accelerometers. Although these devices may have the potential to measure inclination, further work needs to determine their validity and reliability. The potential of a device, such as the AP, which has now been validated for both activity and posture in an adolescent female population, to objectively examine PA, coupled with the objective examination of sedentary patterns and behaviours in free-living populations, is substantial and of great benefit in large-scale health related research.

### Strengths and Limitations

This is the first non-treadmill-based validation study of the activity count function of the AP, which is becoming increasingly popular in PA and health related research. A significant strength of this study was that we employed individualised RMR to normalise energy cost between participants for each activity, rather than the use of standard RMR values [36]. The use of over-ground walking with self-pacing within a particular speed range, which is more effective in simulating real-life activity and reduced the clustering effect that is normally created by using specific speeds, was a significant strength of this study [12]. This study also employed a criterion measures for energy expenditure (indirect calorimetry) while direct observation was used as a measure of posture and activity. The inclusion of sedentary activities and of a range of locomotor activity intensities (light, moderate and vigorous activity) is another strength of this study [12], [33]. Another important strength of this study was the cross-validation of the equation, which was employed to examine the ability of the MR to estimate MET values from accelerometer counts for the AP [12]. An additional strength of the study is the development and cross-validation of activity intensity thresholds which optimise sensitivity and specificity using ROC analysis and the AUC.

This study targeted a specific population, adolescent females, and results from this population may not be generalised to younger children, adolescent males or a wider adult population. High levels of physical inactivity have been observed in adolescent female populations [3], making them a population of great importance when examining activity behaviour, particularly when aiming to implement activity interventions to increase activity levels and decrease sedentary time [43]. The validation of the AP in larger and more variable samples is necessary. The threshold development protocol does not represent the full range of activities undertaken by a population, since it does not include weight bearing and upper body activities [12], [33]. Additionally, during cross validation, the use of different walking speeds over a specified period may be of greater benefit. This methodology may have examined the ability of the thresholds to detect small and quick change in activity intensity. Finally, the use of the low frequency extension may have an effect on the results.

### Conclusion

The accurate and objective examination of PA and sedentary behaviours is critical when establishing links between activity behaviours and indices of health. The AP has previously been identified as a valid and reliable method of directly examining sedentary behaviours. However, the ability of the AP to examine PA patterns through the accelerometer count function has not been investigated. This paper has highlighted high levels of criterion and concurrent validity demonstrated by the AP count function in an adolescent female population, and has also presented optimum thresholds for MPA and VPA in this population. The paper has also highlighted the ability of the AP to distinguish between sitting, standing and stepping time, while also identifying the limitation of the <100 counts min^−1^ sedentary threshold employed by the AG. The findings of this paper support the future use of the AP not only as a measure of sedentary behaviours, but also as a measure of PA in an adolescent female population.
